# Traffic Monitoring via Mobile Device Location

**DOI:** 10.3390/s19204505

**Published:** 2019-10-17

**Authors:** Juan Martín, Emil J. Khatib, Pedro Lázaro, Raquel Barco

**Affiliations:** Communications Engineering Department, Universidad de Málaga, 29630 Málaga, Spain; jmartin@ic.uma.es (J.M.); plazaro@ic.uma.es (P.L.); rbarco@uma.es (R.B.)

**Keywords:** Traffic monitoring system, GPS, map-matching, smartphones, traffic occupancy, smart-city

## Abstract

Measuring traffic in real time is one of the main functionalities of Smart Cities. To reduce the costs of deployment and operation, traffic measurement with mobile devices has been widely studied. In this paper, a traffic monitoring system using mobile devices is proposed. The proposed algorithm has the advantage of having a very low computational cost, allowing most of the pre-processing to be done in the mobile device and therefore making possible the centralized collection of a massive number of measurements. The proposed system is composed of three algorithms; a map-matching algorithm to correct minor location errors, a Virtual Inductive Loop that estimates the traffic and a traffic data collector that aggregates the information from many devices and combines it with other information sources. The system has been tested in a real scenario, comparing its accuracy with a traditional traffic sensor, showing its accuracy.

## 1. Introduction

Nowadays, road networks, and especially cities, suffer from security, ecological and comfort problems due to the massive use of private transport in our daily routine. Traffic congestion, which often follows known patterns, has become an everyday problem in urban environments, resulting in noticeable temporal and economic inconveniences. This has led many cities to enforce certain measures such as restricting which vehicles can be used in a given area based on their license plate number. Smart Cities will deal with this problem in a more sophisticated and less intrusive way, but to be able to regulate the traffic, a basic requirement is to have real time monitoring of the traffic conditions. The traditional methods used by public administrations are fixed measurement devices that collect data about traffic such as inductive loop detectors, pneumatic road tubes, video cameras, laser and other vehicle detectors. Such devices are capable of collecting information like vehicle type, vehicle dimensions or velocity, depending on the type of device used. However, these devices can only collect data on the specific section of road where they are installed. In addition, traffic monitoring infrastructures built with these devices need a heavy investment to develop, deploy and maintain them.

The last few years have seen a dramatic increase in the presence of devices with built in GPS (Global Positioning System) capability. In this work, the focus is on smart phones, which usually have at least two location providers (GPS and the cellular/WiFi network). These devices have two main advantages compared to fixed devices—dynamic coverage area and zero expansion cost.

Mobile phone location data collectors are deemed valid for traffic sensing purposes, since as long as there is a sufficient penetration rate, they will provide accurate measurements of the traffic flow, that is, the sample of users with traffic sensors over the total cars which entered the target road should be at least 2–3% [1,2].

Many researchers have done groundbreaking work in order to make mobile phones, both Global System for Mobile communications (GSM) and Code Division Multiple Access (CDMA) based, practicable as traffic sensors [1,3,4,5,6,7,8,9,10], using either GPS, A-GPS [11], Cell-ID, or Wi-Fi, or a mix of them.

With the increasing growth in the number of mobile devices, the data obtained from them for traffic monitoring has become more reliable. At the same time, this increased reliability comes at a cost in processing power. Traffic monitoring systems must aggregate and process information from a massive number of sensors in real time. This can be considered a Big Data [12] problem, where the volume and the velocity of data generation require special computation strategies. In this case, the high volume of data is due to the large number of devices that report the traffic information. Therefore, in order to increase the capacity, the processing required per report must be minimized. The data reported by these types of sensors is usually a location and a timestamp. Nevertheless, this raw data cannot be used to estimate the traffic data without pre-processing.

The location data must first be associated to a specific road or street. This process is known as map-matching and has a high computational cost. Numerous map-matching methods have been published [13], although not many [14] have proven able to run in real time. Those that do work in real time [15,16,17] are often used extensively in commercial turn-by-turn navigation devices (both dedicated and smart phone based). Some methods are not even disclosed (or only partly disclosed) as they have been patented (e.g., References [9,18]) or become proprietary in nature. The second measurement that is sent with each reading are timestamps. This is used to calculate (among others) the number of users passing through a road per time interval. Again, this information requires some pre-processing and increases the computational cost per measurement. Several publications have proposed data fusion as a way of improving the accuracy of the traffic estimation [19,20,21]. In this paper, the proposed magnitude to easily aggregate data from many sources is the traffic occupancy on a Virtual Inductive Loop (VILD). It is important to point out that traffic occupancy, which measures the time that a specific road segment is occupied by vehicles, should not be confused with vehicle occupancy, which measures the number of passengers per vehicle and is out of the scope of this paper. The main advantage is that this magnitude can be computed in the individual mobile measuring devices and then used in a centralized location to estimate the overall traffic and to fuse it with other data sources [22] with a minimal pre-processing cost.

The main contribution of this paper is the proposal of a practical platform for measuring traffic using mobile devices. The methods that have been proposed in the literature often overlook the computational cost of each location report in the centralized collection point. This drives high server requirements that increase the economic cost of the deployment and reduce its scalability. A reduction of the cost per sample will allow either the use of commoditized equipment (allowing smaller public organizations to use the system) or the scalability of the system into Big Data territory (allowing large geographical areas to be monitored at once). In this paper, the cost per sample is reduced by relying on the mobile devices for most of the computations instead of on the centralized collection point, releasing resources for additional measurements. Specifically, the map-matching and traffic occupancy calculations are executed in the mobile terminals. This, in turn, adds the requirement of light computations that minimize the energy consumption. In this paper, the proposed map-matching algorithm is able to run in real time and at the same time is very light, so it can be run in a mobile device without consuming many resources. For the traffic estimation, the VILD [23] algorithm is used. This algorithm imitates traditional measuring devices, providing the time each measuring device spends at a certain point, that is, the traffic occupancy.

Tests of the platform show the validity of the method and its ability to operate with low sampling rates, which reduces the energy consumption in mobile devices. These tests have been carried out in a real setup, hence validating the algorithm for its practical use in a production environment.

The remainder of this work is structured as follows. Firstly, in Section 2.1, the problems of monitoring device location are presented. In Section 2.2, the proposed solution is described. In Section 2.3, the process of retrieving traffic data from a single vehicle is presented. In Section 2.4, the traffic data combination algorithm is presented. Once the challenges and proposed solutions are shown, these solutions will be tested and the result will be shown in Section 3. Finally, some conclusions about the present work are summarized in Section 4.

## 2. Materials and Methods

### 2.1. Device Monitoring Challenges

In this subsection, two typical challenges in traffic monitoring systems via location devices are described: inaccurate location and path estimation.

#### 2.1.1. Inaccurate Location

In this paper, the location needed in traffic monitoring is provided by two sources—GPS and the network provider. Both elements involve error in the estimated location.

On the one hand, in GPS systems, the main error source is due to inaccurate time-keeping by the clock of the receiver device. Microwave radio signals traveling at the speed of light from at least three satellites are used to calculate the device position, altitude and velocity. The receiver and satellite clock error components are multiplied by the speed of light *c*. Hence, because of the factor *c*, a small clock error can cause a very large code and phase error. For example, a clock error of 1 ns translates into 0.3 m in range error, whereas 1 μs implies an error of 300 m. Increasing the receiver clock precision by atomic clocks is expensive so it is not a feasible option for commercial devices like smart phones.

On the other hand, the network location provider uses WiFi hotspots and cell towers known to the Android device to approximate the location of a user. When the location provider is polled, the IDs of the WiFi hotspots and cell towers in the area are sent via Internet to the Google Location Server, a database with location information on WiFi hotspots and cell towers. The Google Location Server returns the approximate location of the user. WiFi hotspots allow an accuracy of 100 m–500 m, whereas cellular networks only allow an accuracy greater than 500 m. This means the network provider will be very inaccurate in areas without WiFi hotspots.

So, whatever the location provider is, the error must be taken into account in order to estimate the real location of the user.

#### 2.1.2. Path Estimation

The mobile phone can be programmed to periodically send location reports. However, a minimum or constant frequency for the location report is not guaranteed, due to different problems like coverage holes (on network or on location provider), network congestion or user data tariff. Specifically, the challenge at this point is achieving an acceptable accuracy in map-matching with a low frequency sampling. The lower the location sampling data the higher the difficulty of the map-matching problem. On the other hand, a low frequency sampling minimizes battery consumption and network usage.

The map-matching problem (described in more detail in Section 2.2) can be defined as finding the path that links a sorted list of locations on a road network and that is the most probable followed trajectory.

With large distances between consecutive locations the difficulty for map-matching is higher because there are more possible trajectories that connect these consecutive locations, therefore there are more possible trajectories between the first and the last location.

### 2.2. Proposed Solution

**Figure 1:**
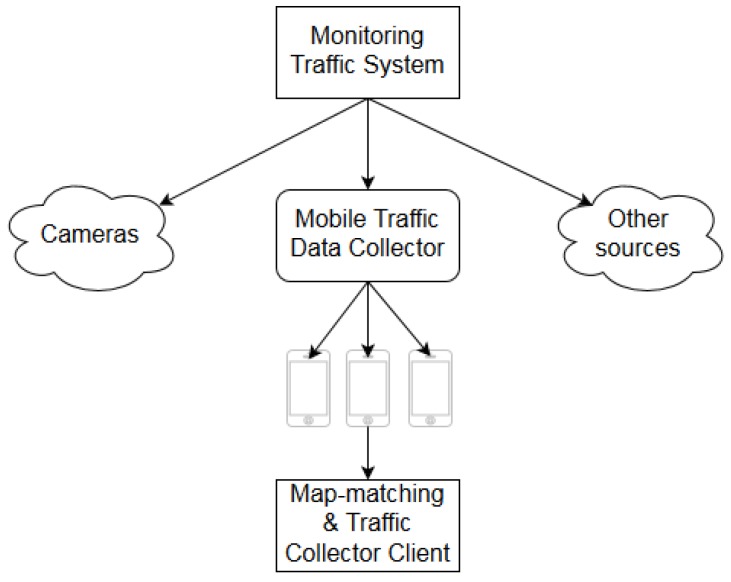
Architecture of traffic monitoring via mobile devices.

In this paper, a centralized system to collect traffic status data from several sources is proposed. Figure 1 shows the architecture of the system. The main module that aggregates data from several data sources is the Monitoring Traffic System. Table 1 shows the data sources that report measurements directly to this module. In Section 2.4 a common format for the data sent to the Monitoring Traffic System is defined. This common format will greatly reduce the processing required per sample, thus increasing the capacity of the main module. The requirements and implementation details to adapt the data sources described in Table 1 to the proposed format, as well as the internal structure of the Monitoring Traffic System are out of the scope of this paper. An additional module, the Mobile Traffic Data Collector, collects all the measurements from individual mobile sensors and aggregates them obtaining a measurement that is equivalent to the traffic sensors in Table 1. The Mobile Traffic Data Collector is connected to each mobile sensor in a typical client-server architecture:

- **Client**: Contains the functionalities of map-matching and traffic data collection, relieving the server of these tasks. The mobile application is responsible for tracking the device on a journey. The application receives the device location from the selected location provider (i.e., GPS, mobile network, etc.) and keeps the two latest locations. With these latest locations the application applies map-matching in order to minimize the location error and associate it with a road (explained in detail in Section 2.3.1). These corrected locations are used to estimate the followed route with the algorithm described in Section 2.3.2. Then, in each VILD belonging to the estimated route, the time when the mobile device crossed it (time-stamp) and the time spent are computed. The computed timestamps are joined to the time needed to cross each VILD. Finally, these traffic data are reported to the server.
- **Server**: The server side software aggregates the data from all the mobile devices. This task is described in detail in Section 2.4 following the execution flow shown in Figure 7. Traffic data packets sent by the client application are received through a web Application Programming Interface (API). The data is added to the traffic data window in the collector module where it waits to be computed in its corresponding time-slot. Finally, the result of the combination of data in each time-slot is sent to the Monitoring Traffic System where it can be fused with data from other sources.

### 2.3. Single Car Location

Reported location and VILDs will rarely be at the same point, due to different factors such as the location error or the frequency of location measurements. Thus, the route followed by a passenger in the car will be calculated with map-matching. Once the route is known, it is possible to derive when the user went through a measurement point (VILD). In the event that more than one device in the vehicle reports its location, duplicated VILD crossing reports may occur. Since the devices have no way of knowing about each other, the detection of such duplicities is up to the server.

#### 2.3.1. Map-Matching

**Figure 2:**
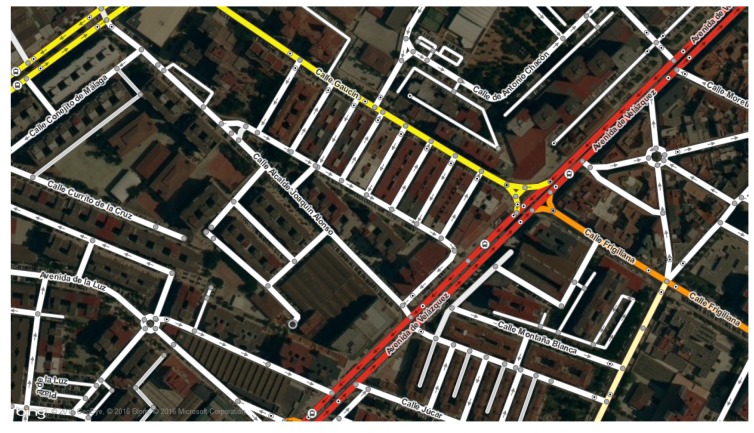
Digital map with graph representation.

Map-matching is the process of associating a sorted list of locations to the road network on a digital map. A digital map is represented by a mathematical graph *G* as shown in Equation (1). A graph is a representation of a set of objects or vertices where some pairs of these objects are connected by links. These objects are typically called vertices or nodes and the links that connect them are called edges or arcs. Figure 2 shows a map area on graph representation.

(1) $$\begin{array}{cc} G & =(V,E)\\ V & =\{v_{1},v_{2},\ldots \},V\;\mathrm{is}\;\mathrm{the}\;\mathrm{set}\;\mathrm{of}\;\mathrm{vertices}\\ E & =\{\left(v_{i},v_{j}\right)|v_{i},v_{j}\in V\},E\;\mathrm{is}\;\mathrm{the}\;\mathrm{set}\;\mathrm{of}\;\mathrm{edges} \end{array}$$

The aim of a map-matching algorithm is to reconstruct from a location sequence the path driven by a vehicle on the road network. The main difficulties in this task derive from the errors of location providers positioning measurements and from the uncertainty introduced by the sampling of the data.

Most recent works related to map-matching are focused on solving, with ever more accuracy and reliability, the same problem of keeping track in real-time of the correct position of the user on a map. Thus, the positioning data used for this kind of task usually has a high sampling frequency (∼1 sample per second, 1 Hz). Many different techniques have been developed to solve this kind of map-matching problem, ranging from simple geometrical considerations to more advanced inference methods but they are commonly categorized for simplicity into four groups—geometric, topological, probabilistic and advanced [24]. In this paper, a geometric based approach is followed.

The lower complexity of this kind of map-matching algorithm allows it to run smoothly on a smartphone. When the user is traveling by car, the tracked phone is located in the car too, so location provider coverage is often low and, therefore, the location error is normally high. In addition, it must be taken into account that the sampling frequency may not be high. High sampling frequency drains the battery of the smartphone and might be costly depending on the data tariff. Thus, it is necessary to find a compromise between accuracy, computational cost and battery consumption.

**Figure 3:**
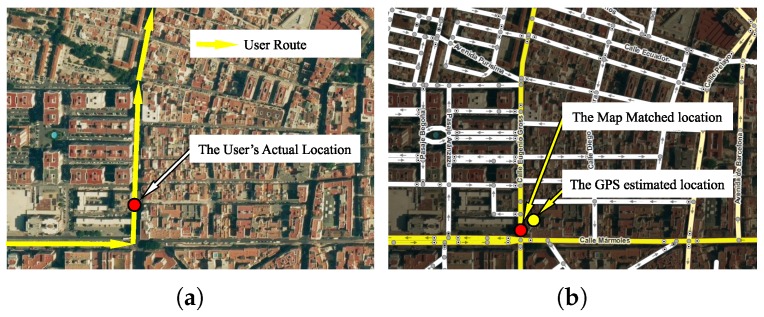
The map-matching problem. (**a**) Map of actual streets; (**b**) Digital Map and map-matching result.

Figure 3 illustrates the map-matching problem. Figure 3a represents the user’s route and his actual location on the street. Figure 3b shows the location estimated by location provider and the map-matching location result. As illustrated in Figure 2 and Figure 3b, a network representation consists of a set of straight lines in $R^{2}$, called arcs. An arc is an edge in graph definition (1), and each arc endpoint corresponds with a vertex. In map-matching theory these vertices are called nodes. Nodes also represent the junctions between two or more arcs (street intersections).

**Figure 4:**
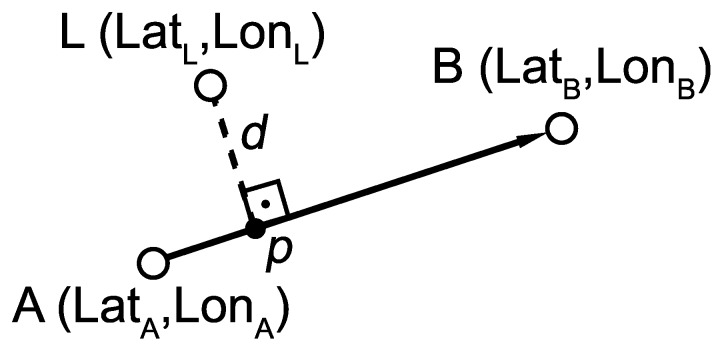
Distance between Location and AB arc.

The proposed algorithm works with the last two locations and the digital map as inputs. The first task is to find the nearest arcs in the map for each location. The algorithm computes the distance between the locations and each arc. Figure 4 and Equation (2) describe the process of this task where *L* is the current location and AB represents the arc. The arcs with distance below a threshold (in our case 5 m) are added to the list of possible traveled arcs. This list is sorted by distance in ascending mode. The outputs of this algorithm are two sorted lists in ascending order, one list with the possible arcs for the last location, and another one with the possible arcs for the previous location. These lists are the inputs to the algorithm described in Section 2.3.2:

(2) $$\begin{array}{cc} \vec{dir} & =B-A\\ \vec{diff} & =L-A\\ proj & =\frac{\vec{dir}\cdot \vec{diff}}{{\left|dir\right|}^{2}}\\ p & =\left\{\begin{array}{cc} A & if\;proj\leq 0\\ B & if\;proj\geq 1\\ A+proj\cdot ({\vec{dir}}_{x},{\vec{dir}}_{y}) & otherwise \end{array}\right.\\ d(L,AB) & =d(L,p) \end{array}$$

where:

*A*: one endpoint of arc
*B*: another endpoint of arc
*L*: location from location provider
d(): distance between two points

#### 2.3.2. Estimated Route

**Figure 5:**
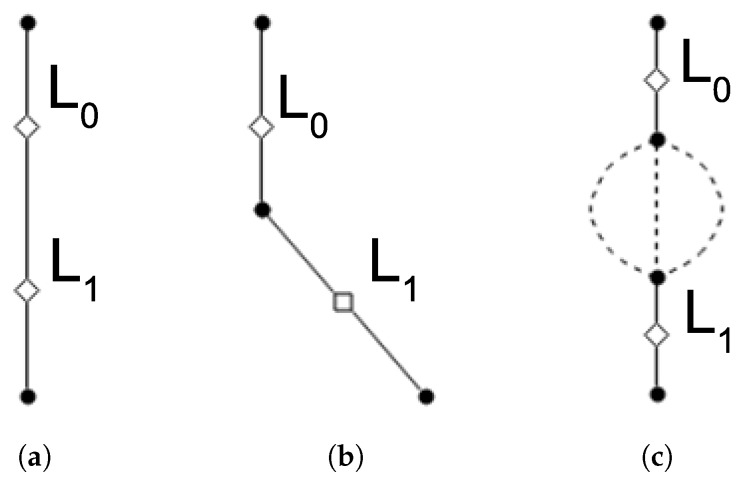
Different situations depending on locations. (**a**) Two locations in the same arcs; (**b**) Two locations in neighboring arcs; (**c**) Two locations in non-neighboring arcs.

In order to decrease battery consumption the localization frequency must be low. However, a low frequency in the localization process entails three types of situations which are shown in Figure 5. The inputs to this algorithm are two arcs and their respective locations. The three possible situations are as follows:

- Two locations in the same arc: this trivial case occurs when the two estimated locations are in the same arc as shown in Figure 5a.
- Two locations in neighboring arcs: this case involves a transition between two linked arcs as illustrated in Figure 5b.
- Two locations in non-neighboring arcs: this is the most complex case and it adds uncertainty to the process of estimating the route followed by user. In Figure 5c we can see that there are several arcs between the two selected arcs.

**Figure 6:**
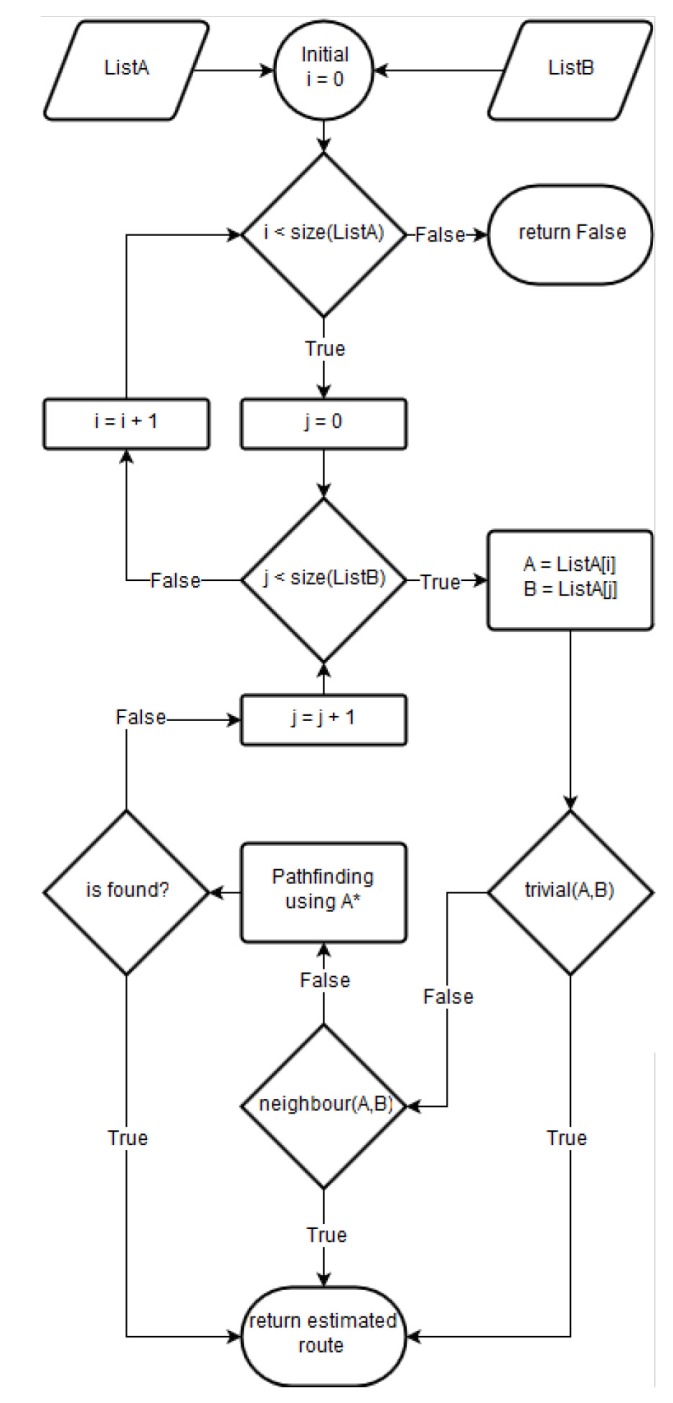
Flow diagram of the route estimating algorithm.

Figure 6 describes the proposed algorithm. The three branches work as follows:

1. Trivial case: the algorithm returns any of the two input arcs, since they are the same.
2. Two arcs: the algorithm returns a list with the first and second input arcs. The first arc is directly linked with the second because the user has just visited these arcs in the last time slot.
3. Multiple path options: when there is one or more arcs between the input arcs, the algorithm executes an implementation of the A* path-finding algorithm [25]. The execution returns the shortest path between the input arcs or false if there is not a solution.

Finally, the algorithm returns the estimated route or false if there is no path between the two input arcs. This algorithm is executed each time a new location measurement is obtained.

#### 2.3.3. Virtual Inductive Loop Detector

In order to measure the traffic status, once the route traveled by the user and the spent time (difference time between the last location and the previous location) are known, a Virtual Inductive Loop detector (VILD) technique is proposed. The VILD is a segment of an arc of a digital map, which simulates the operation or a real Inductive Loop Detector. When a VILD is crossed by the user device, the application in the device composes a traffic data packet to send to the Mobile Traffic Data Collector, as explained in Section 2.4. This avoids sending superfluous data to the server, optimizing the processing capacity of the system. The traffic data packet includes the measurement time stamp, occupancy, list of traveled arcs, time spent and traveled distance, although only the occupancy is used to estimate traffic in the Monitoring Traffic System. The rest of the data is saved for possible future uses.

During the implementation, it has been observed that the location provider error implies bad locations causing inaccurate measurements in the following cases:

- Vehicle is stopped and location is shown unstable. This problem is solved requiring a minimum distance between the new and the old location.
- When the time interval between the two locations is very high (e.g., due to lost signal), the estimated route in Section 2.3.2 is probably unacceptable. So, when there is a large time interval between the new and old location, the old location is discarded.
- Anomalous speed Equation (3) estimation may occur when the estimated route between the two last locations is too large or when the time interval between these locations is too long. To preserve the system from these anomalous data, speeds above a certain threshold are discarded. If true high speed cases were discarded, it will not have a big impact on traffic monitoring because high speeds are not related to traffic congestion.
  (3) $$\begin{array}{cc} speed & =\frac{distance\;traveled}{time\;spent} \end{array}$$

### 2.4. Traffic Data Collector

**Figure 7:**
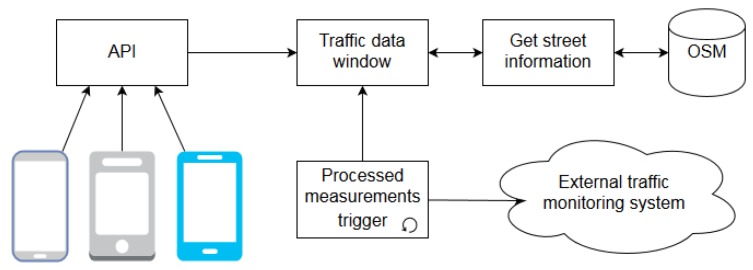
Traffic data collector system.

Reported data by a single user are not robust enough to estimate city traffic. A single driver could stop for different reasons (e.g., pedestrian at zebra-crossing, parking his car, etc.). However, if the reported data from all users in each arc at a given time interval are combined, a better approach to the traffic conditions can be obtained. The proposed system for traffic data combination, included in Traffic Data Collector is shown in Figure 7. Firstly, the client application sends the traffic data calculated according to Section 2.3.3 to the system through a web API in Figure 7. Secondly, these data are pushed in the Traffic Data Window where they are combined with data from other users in the same arc and time-slot, as explained in Section 2.4.1. The required current arc information (e.g., number of lanes) for this process is requested from Open Street Map (OSM [26]). OSM is an open read and write access platform about street maps data. Concurrently, there is a trigger module responsible for transmitting the result of the combination data to an external traffic monitoring system (system that works with heterogeneous traffic sensors network).

It is also in this point where the duplicate entries (i.e., those where different devices report the same VILD with very similar timestamps) should be filtered. This can be done with two filtering rules depending on the number of lanes in the VILD:

- The duplicated reports are done on a single-lane VILD: both will have the same or very similar timestamp; hence, only the first report to reach the traffic data collector will be considered.
- The duplicated reports are done on a multiple-lane VILD: this situation is more complex, so once two reports have the same timestamp, a verification process must be launched to check if they are located in different vehicles. This verification process will check if the last $N_{R}$ reports (where $N_{R}\geq 2$ is a configurable value) of the devices are also duplicates. If this is the case, only the first report is considered.

#### 2.4.1. Traffic Data Window

**Figure 8:**
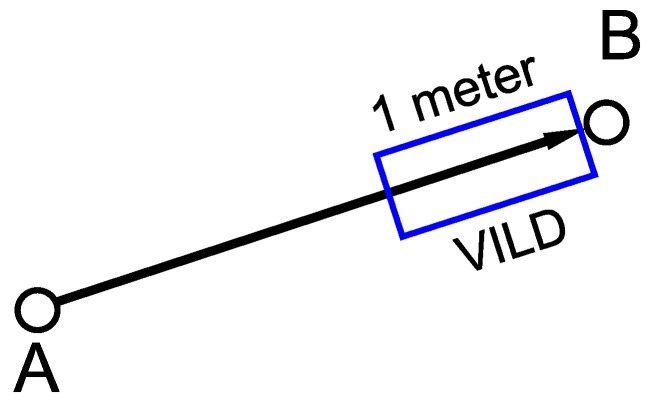
Virtual Inductive Loop Detector (VILD) on AB arc.

Tracking a single device (smartphone) is unreliable because it is affected by single user decisions. However, if most drivers stop at a given street, the most probable cause is a traffic jam. Therefore, it is essential to have a large number of drivers in each street. The combination process aggregates data in two ways:

- Spatial: this combination is relatively simple, grouping the traffic measurements by arc. However, users can be in the same arc but at different points. Therefore, the same measurement point should be defined for each user. Usually, real inductive loop detectors have a length between 1 and 2 m, in this work, the length of the VILDs are set to 1 m, and they are located at the end of the arc. The VILD location in the arc is not important because there are not intersections inside the arcs. Figure 8 illustrates a 1 m VILD located at the end of arc AB.
- Temporal: the flow of data reception is not continuous so it is important to use a window that groups the data in time slots. This combination contributes to the measurement robustness by avoiding abrupt variations caused by exceptional situations such as a driver parking the car or waiting at a zebra crossing.

**Figure 9:**
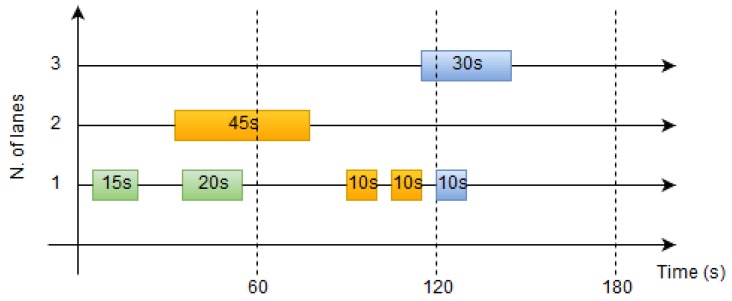
Window collector.

Figure 9 shows the state of the window collector data in a 3 lanes road along 180 s. In this snapshot the window has 7 traffic data packets from anonymous drivers. The time-slot size is set to 1 minute which is enough to minimize abrupt variations while providing fresh measurements. In Table 2, the traffic intensity and occupancy for each time-slot the output of the collector are computed with the measurements that have finished before the time slot ends.

#### 2.4.2. Traffic Data Estimation

There is no standardized definition for traffic congestion [27]. Therefore, there are different equally valid measurements in order to study the traffic congestion [28]. In this paper, the following normalized traffic indicators are chosen:

(4) $$\begin{array}{cc} I(veh/hour) & =vehicles_{ij} \end{array}$$

(5) $$\begin{array}{cc} O(\%) & =\frac{\sum Tveh_{ij}}{\text{time-slot}\_length}100 \end{array}$$

In Equation (4) *I* is the intensity of the traffic in a specific segment of the road at one time-slot, $vehicles_{ij}$ are the number of vehicles inside VILD *i* and time-slot *j*. In Equation (5) *O* is the traffic occupancy in a specific segment of road (VILD), where $Tveh_{ij}$ is the time that the vehicle is inside the VILD (*i*) at time-slot *j* and time-slot_length is the length of the time-slot used (60 s in this work).

A VILD measures the traffic over all lanes of a road (except lanes for special use, e.g., a bus lane). The number of lanes in each road is different so it is important to normalise these measurements to the number of lanes. Table 2 shows the value of these indicators at different time-slots taking into account the number of lanes in the example in Figure 9.

Finally, intensity and traffic occupancy are reported by the Processed measurements trigger module to the external traffic monitoring server. These indicators in particular are very useful because they allow to compare, even complete the traffic estimation reported by different measuring devices (e.g., vision-based systems [22]). Nevertheless, it is important to consider that the intensity is just a partial measurement, since it only reflects the intensity of vehicles that have a measurement device, which is just a sample of the total. This implies that the absolute value of the intensity will not necessarily be useful, but its general behavior (growth or reduction) may be of interest for some applications. In addition, it is possible to get these indicators from other sources with simple transformations.

## 3. Results and Discussion

To test the entire solution shown in this work, a set of tests has been defined, covering different modules of the proposed system. The data used during the experiments was collected from Málaga city with a Samsung Galaxy Note 8.

In this section, first the performance of the map-matching method presented in Section 2.3.1 is evaluated with different location sampling frequencies. Then, the traffic estimation accuracy is analyzed by comparing the returned data with the data reported by the pneumatic road counter MetroCount^®^ *Roadside Unit* MC5600.

### 3.1. Map-Matching Location Frequency

The aim of the following experiment is to demonstrate the performance of map-matching using the route estimator algorithm. The experiment relates the accuracy in the estimation and the frequency used in location requests. Map-matching is focused on the correction of the intrinsic location error and fitting the location to the route. The route estimation is focused on minimizing the error provided by the lack of data location. Although it seems evident that high location sampling frequencies can achieve more accuracy in the route estimation, this improvement may be less meaningful in the map-matching performance due to the intrinsic error by location providers.

For the execution of this experiment, the location provider was GPS and the range of location frequency updates was established between 1 Hz and 0.016 Hz (a period between 1 s and 60 s). During the tests, the device went over 100 Km in different types of regions and with variable speed—near high buildings, areas without buildings, streets, highways and traffic jams.

**Figure 10:**
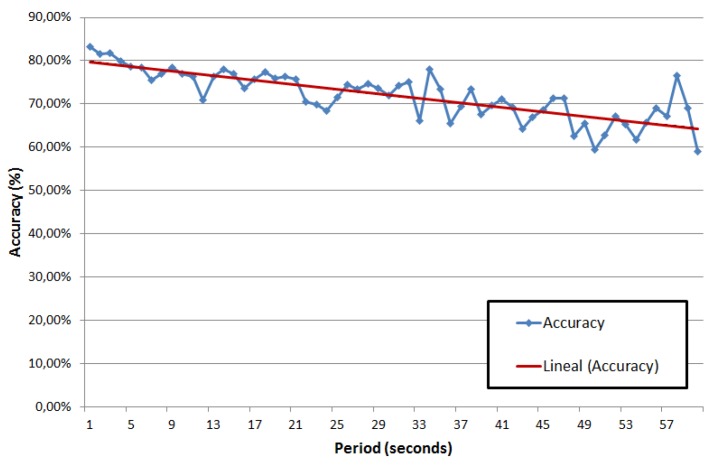
Map-matching accuracy over frequency.

The results of this experiment show that the average accuracy Equation (6) is in the range of 60% to 85% as shown in Figure 10. The accuracy of estimated route tends to a lineal equation proportional to the location sampling frequency.

(6) $$\begin{array}{cc} Accuracy(\%) & =\frac{number\;of\;correct\;arcs}{total\;arcs\;in\;route}\cdot 100 \end{array}$$

As it can be seen from the results, the accuracy is worse with the high than with the low period between samples. This is primarily due to the increase of the possible routes when the first and last point are far from each other. When the period between samples is low, the distance between the measurements is also low, and the number of possible alternative routes among them is usually just one. On the other hand, when the period is high, the distances between the measurements may be longer (depending on the speed) and hence the number of possible routes may be more than one, thus increasing the number of errors.

The results show an upper limit of 85% in accuracy when the samples are taken every second. This limit is due to the intrinsic error provided by the GPS location system; as described in Section 2.1.1, although the location is provided every second this location may be wrong due to the GPS error and so the estimated route will be wrong too. This matching rate range is similar to the result of the algorithm proposed in [14] which needs samples every second.

Overall, the correct sampling frequency must be chosen considering a trade off between accuracy and energy saving. A higher sampling frequency will provide a higher accuracy (limited only by the accuracy of the location provider), at the cost of a larger number of executions of the map-matching algorithm. A lower frequency will decrease the total processing time, therefore reducing the energy consumption of the device. Given the results shown in Figure 10, it can be concluded that the sampling frequency can be reduced several times without much loss of accuracy; for instance, a reduction from 1 Hz to 0.1 Hz will reduce the battery consumption by 10, while the loss of accuracy is only by 5%. It is important to notice that in this trade off, the processing capacity of the server (that is, the Mobile Traffic Data Collector and the Monitoring Traffic System) is not affected, since it only receives information when a VILD is crossed.

### 3.2. VILD Versus Validated Device

In order to verify the accuracy of the solution, in this experiment it is compared with a validated traffic monitoring device. The reference device is a pneumatic road counter model *MetroCount^®^ Roadside Unit MC5600*. This device is able to register the traffic in both ways. MC5600 reports multiple traffic data statistics but in this test only the traffic occupancy and the intensity have been selected. These will be the indicators to determine the system accuracy.

**Figure 11:**
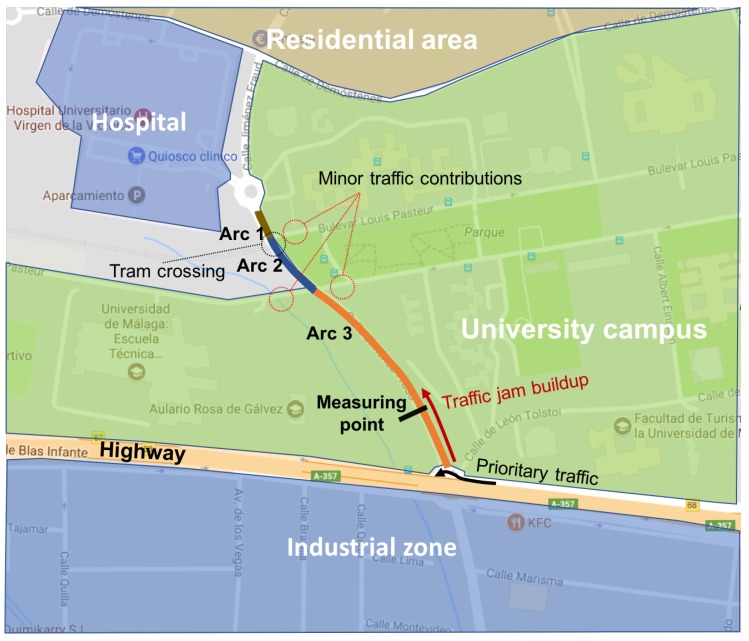
Measurement scenario.

The device was installed in Jiménez Fraud street at location=(36.715151,−4.474060). Figure 11 shows the MC5600 location and the route followed during the test. This street is the main traffic connection between a highway and the University of Málaga campus. It also serves a hospital and a residential area. The monitored part covers a section between two roundabouts. The northern end connects with the hospital and the residential area, and the southern edge with a major industrial complex. The street is also crossed by a tram line that cuts the traffic every 10 mins, and by two minor streets that add a negligible amount of traffic. This street has a traffic pattern that repeats daily: at office hours, the intensity and traffic occupancy are very high, with traffic jams at the peak hours (beginning and end of the office hours) where cars stop for several minutes; outside of office hours, the intensity is very low, with cars stopping for some seconds occasionally for pedestrians or to yield on the roundabouts or at the tram crossing. MC5600 was reporting traffic data every hour during 17 days in both ways. Mobile devices with the implemented software were reporting at the same time but with blank time periods due to the schedule of testing users; no testing was done at night, although in this location the traffic is less significant at night. Besides, the number of VILD reports each hour is not constant because the traffic intensity changes along the day. So, in order to test the solution with a constant number of reports, the data set in each hour is filled to 60 (one sample per minute) samples per hour using stochastic regression with a uniform noise of ±10%.

In this test, the estimated traffic occupancy provided by the VILD method is studied. However, the study of the absolute value of the intensity is useless because it is estimated by counting the devices connected to the system in each arc, not the total number of vehicles. For example, if 60 vehicles travel along some road in one hour but only 30 of them are connected to the system, then the VILD reports an intensity of 30 vehicles per hour instead of 60 vehicles per hour. However, the trend of this partial intensity may reflect the general behavior of the real intensity, especially if the rate of the vehicles that have a measuring device is known.

**Figure 12:**
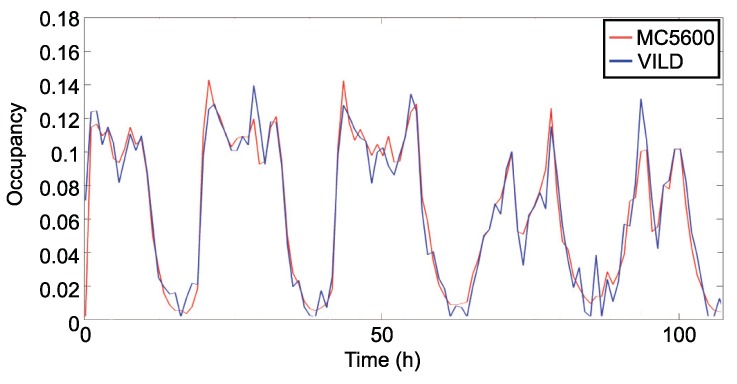
Traffic occupancy with 60 samples per hour.

**Figure 13:**
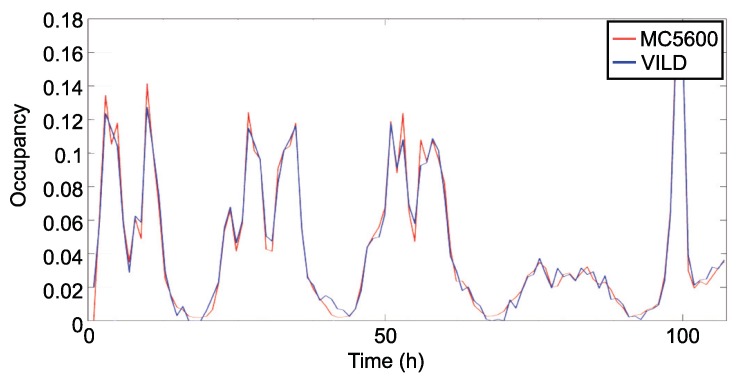
Traffic occupancy with 60 samples per hour (opposite direction).

Figure 12 and Figure 13 represent the occupancy every hour in the monitored road in both directions. This representation is produced with 5 days of samples. The traffic occupancy provided by VILD is estimated with 60 measures per hour in each way of the road. In both figures, we can see that the estimated occupancy is near to the occupancy provided by MC5600. This trend continues for the rest of samples collected during the experiment.

**Figure 14:**
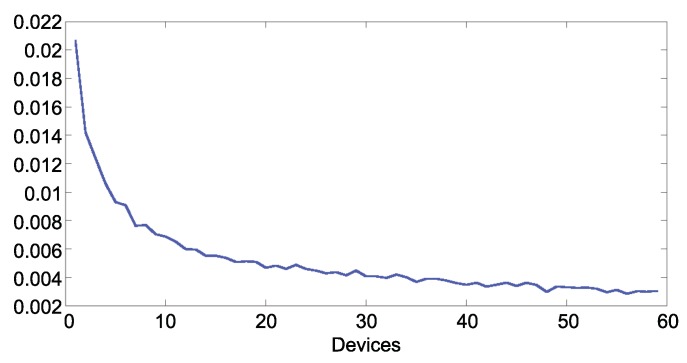
MSE of the traffic occupancy per samples per hour.

**Figure 15:**
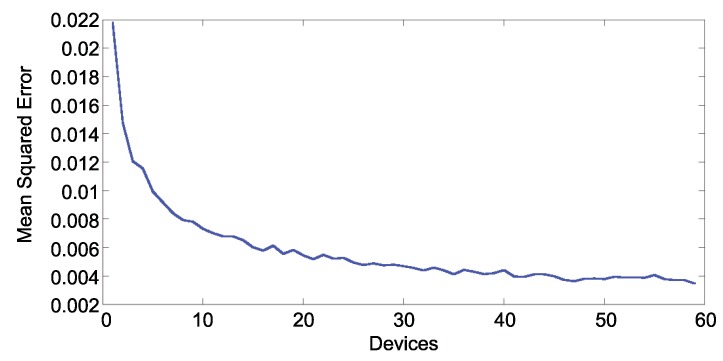
MSE of the traffic occupancy per samples per hour (opposite direction).

Figure 14 and Figure 15 show the error in estimated traffic occupancy with different numbers of devices. As shown in both figures the mean square error is inversely proportional to the number of devices reporting. With 10 samples per hour the algorithm reduces the occupancy error to 0.007. The error with less samples is not acceptable because it has the same magnitude of the measurement itself.

## 4. Conclusions

In this paper, a complete system for monitoring traffic status using smartphones has been presented. The performed tests show the accuracy and effectiveness of the proposed approach in estimating routes and traffic occupancy. In these tests, the reported estimated route is compared with the true followed route and the occupancy in a road with the measurements of the validated traffic monitoring device MC5600.

In the performed tests, the map-matching algorithm has proven a high accuracy with different sampling frequencies, taking into account the error provided by the GPS location. The results of map-matching show correct matching rate between 66% and 86% with samples every minute and every second respectively.

The traffic monitoring system has shown a high accuracy of the occupancy time estimation. The intensity of the traffic has not been tested because the estimation is proportional to the number of devices reporting in each road as mentioned above. This aspect is hard to improve if only the proposed method is used and there are many devices not transmitting any information. A solution, proposed for future work, is to combine the information from the mobile devices with data from other traffic measurement sources. The traffic occupancy estimation in the experiments has demonstrated that at least 10 vehicles reporting in one hour are required to maintain confidence in the results.

Future lines of research will involve improving the map-matching results and proposing algorithms to estimate via mobile devices the number of vehicles traveling.

## Figures and Tables

**Table 1:** Most commonly used traffic vehicle detectors.

Device	Advantages	Disadvantages
Inductive Loop	Supports all weather and lighting conditions	Intrusive installation and high maintenance
Pneumatic counter	Portable and non-complex installation	Damaged by vehicles
Cameras	Non-intrusive installation and flexible set-up	Inclement weather, shadows, poor-lighting

**Table 2:** Traffic measures from window collector.

Time (s)	Intensity (veh/h)	Occupancy (%)
60	$\frac{2\cdot 60}{3}=40$	$\frac{15+20}{3\cdot 60}=25\%$
120	$\frac{3\cdot 60}{3}=60$	$\frac{10+10+45}{3\cdot 60}=36.1\%$
180	$\frac{2\cdot 60}{3}=40$	$\frac{10+30}{3\cdot 60}=22.2\%$
